# Supplementary material for: Nucleophosmin 1 promotes mucosal immunity by supporting mitochondrial oxidative phosphorylation and ILC3 activity
Source: Nat Immunol. 2024 Aug 5;25(9):1565–79. doi: 10.1038/s41590-024-01921-x (PMC11362010; doi:10.1038/s41590-024-01921-x)
Supplement: Supplementary file 1 — Supplementary Figs. 1 and 2 and Supplementary Tables 1 and 2. [file 41590_2024_1921_MOESM1_ESM.pdf]

nature immunology

Supplementary information https://doi.org/10.1038/s41590-024-01921-x

Nucleophosmin 1 promotes mucosal immunity by supporting mitochondrial oxidative phosphorylation and ILC3 activity

In the format provided by the authors and unedited

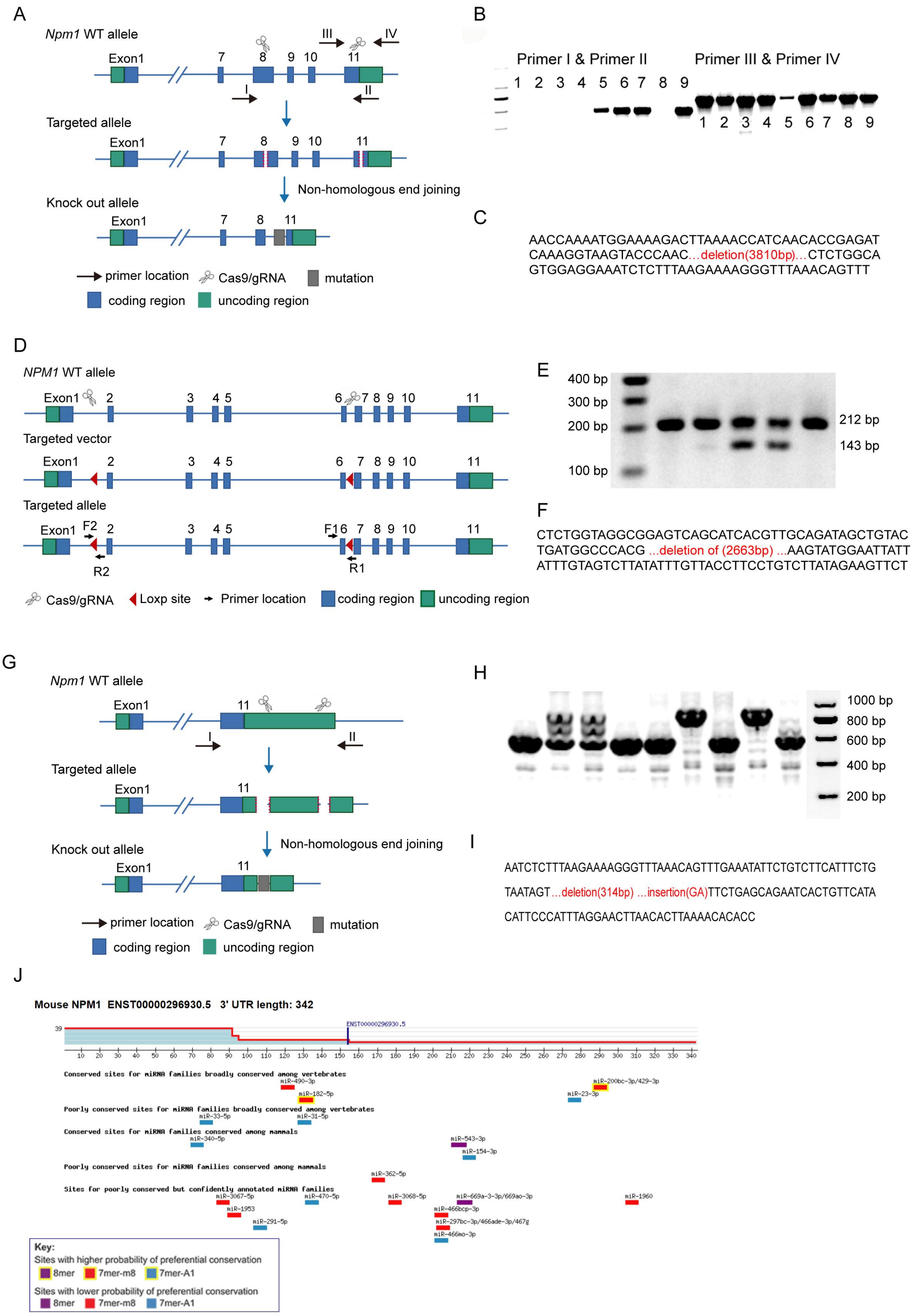

Supplementary Fig.1. Generaion of *Npm1*$^{+/-}$, *Npm1*$^{flox/+}$ and *Npm1*$^{UTR+/-}$ mice using CRISPR/Cas9 technology.

(A) Schematic illustration for *Npm1*$^{+/-}$ mice of Cas9/gRNAs (guide RNAs)-mediated gene-targeting for the *Npm1* genomic locus indicating the locus, the targeted allele and knock-out allele.

(B) PCR (location of primer I to IV showed in A) was employed to genotype *Npm1*$^{+/-}$ or WT mice carrying WT allele (679 bp, right) and/or knock-out allele (521 bp, left).

(C) Sanger sequencing of knock-out allele showed that *Npm1*$^{+/-}$ mouse carries a deletion of 3810 bp.

(D) Schematic illustration for *Npm1*$^{flox/+}$ mice of Cas9/gRNAs (guide RNAs)-mediated gene-targeting for the *Npm1* genomic locus indicating the locus, the targeted allele and knock-out allele.

(E) PCR (location of primer I to II showed in D) was employed to genotype *Npm1*$^{flox/+}$ or WT mice carrying WT allele (143 bp, right) and/or loxp sequences flanked allele (212 bp, left).

(F) Sanger sequencing of knock-out allele showed that *Npm1*$^{flox/+}$ mouse carries a deletion of 2663bp when crossed with *Rorc*$^{cre/+}$ and *Villin*$^{cre/+}$.

(G) Schematic illustration for *Npm1*$^{UTR+/-}$ mice of Cas9/gRNAs (guide RNAs)-mediated gene-targeting for the *Npm1* genomic locus indicating the locus, the targeted allele and knock-out allele.

(H) PCR (location of primer I to II showed in F) was employed to genotype *Npm1*$^{UTR+/-}$ or WT mice carrying WT allele (679 bp, upper band) and/or knock-out allele (521 bp, lower band).

(I) Sanger sequencing of knock-out allele showed that *Npm1*$^{UTR+/-}$ mouse carries a deletion of 314 bp and an insertion of GA on its 3'UTR region.

(J) The microRNAs binding sites on the deleted UTR region were predicted by TargetScan.

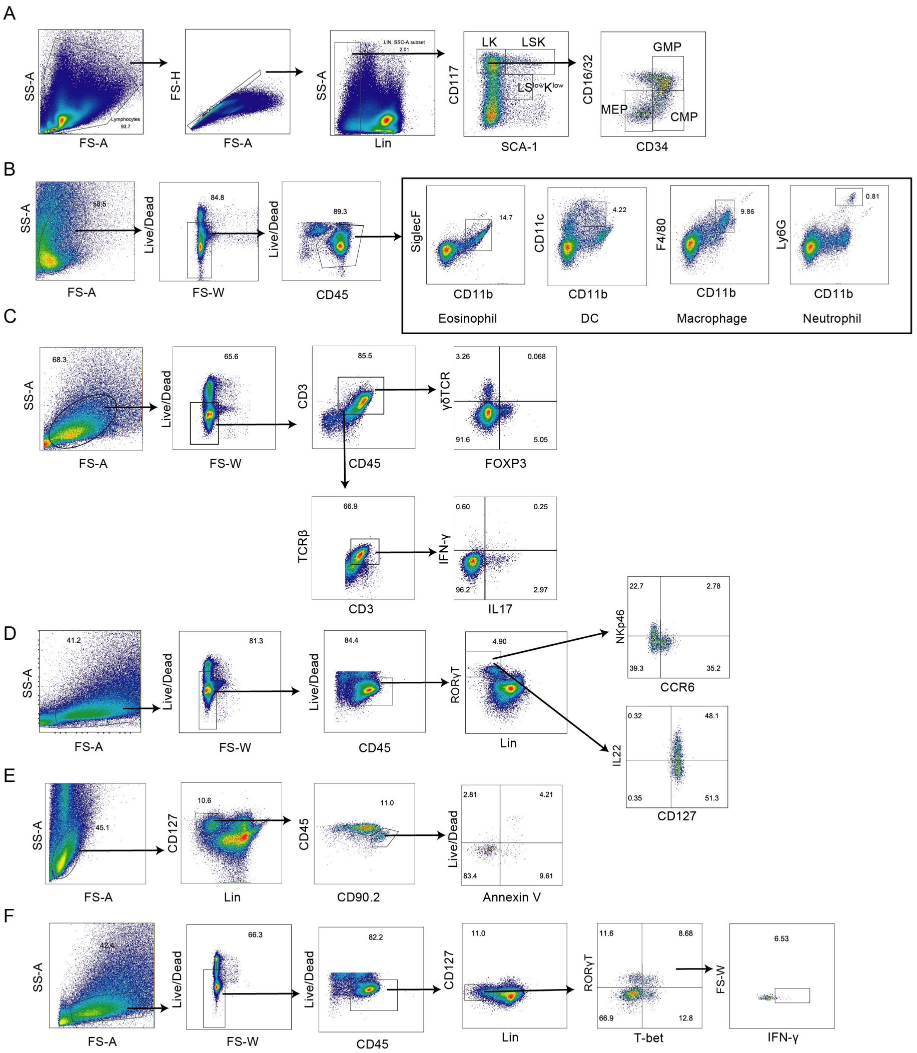

Supplementary Fig.2. Gating strategies for flow cytometry data.

(A) Gating strategy for LK, LSK, $LS^{low}K^{low}$ ($Lin^{-}Sca1^{low}Kit^{low}$), MEP, GMP, and CMP populations in mouse bone marrow cells.

(B) Gating strategy for eosinophils, dendritic cells (DCs), macrophages and neutrophils from lamina propria lymphocytes (LPLs).

(C) Gating strategy for Treg, γδT and Th17 from LPLs.

(D, E) Gating strategy for ILC3 cells from LPLs to analysis its subsets and cytokines secretion (D) and apoptosis (E).

(F) Gating strategy for IFNγ+ ILC3s from LPLs.

Supplementary Table 1. Basic characteristics of patients in this study.

Donor	Age	Gender	Samples
UC1	50-60	Male	Rectal biopsy sample
UC2	60-70	Male	Rectal biopsy sample
UC3	40-50	Male	Rectal biopsy sample
UC4	30-40	Male	Rectal biopsy sample
UC5	50-60	Male	Rectal biopsy sample
UC6	50-60	Male	Rectal biopsy sample
UC7	50-60	Male	Rectal biopsy sample
UC8	40-50	Male	Rectal biopsy sample
UC9	40-50	Male	Rectal biopsy sample
UC10	50-60	Male	Rectal biopsy sample
UC11	60-70	Male	Rectal biopsy sample
UC12	60-70	Male	Rectal biopsy sample
UC13	30-40	Male	Rectal biopsy sample
UC14	30-40	Male	Rectal biopsy sample
UC15	40-50	Male	Rectal biopsy sample
UC16	40-50	Female	Rectal biopsy sample
UC17	40-50	Female	Rectal biopsy sample
UC18	30-40	Female	Rectal biopsy sample
UC19	40-50	Female	Rectal biopsy sample
UC20	50-60	Female	Rectal biopsy sample
UC21	50-60	Female	Rectal biopsy sample
UC22	50-60	Female	Rectal biopsy sample
CD1	50-60	Male	Rectal biopsy sample
CD2	40-50	Male	Rectal biopsy sample
CD3	40-50	Male	Rectal biopsy sample
CD4	30-40	Male	Rectal biopsy sample
CD5	50-60	Male	Rectal biopsy sample
CD6	50-60	Male	Rectal biopsy sample
CD7	40-50	Male	Rectal biopsy sample
CD8	40-50	Male	Rectal biopsy sample
CD9	50-60	Male	Rectal biopsy sample
CD10	40-50	Male	Rectal biopsy sample
CD11	50-60	Male	Rectal biopsy sample
CD12	30-40	Male	Rectal biopsy sample
CD13	40-50	Female	Rectal biopsy sample
CD14	40-50	Female	Rectal biopsy sample
CD15	40-50	Female	Rectal biopsy sample
CD16	50-60	Female	Rectal biopsy sample
CD17	50-60	Female	Rectal biopsy sample
CD18	40-50	Female	Rectal biopsy sample
CD19	30-40	Female	Rectal biopsy sample
CD20	40-50	Female	Rectal biopsy sample

Ctrl1	30-40	Male	Rectal biopsy sample
Ctrl2	30-40	Male	Rectal biopsy sample
Ctrl3	20-30	Male	Rectal biopsy sample
Ctrl4	30-40	Male	Rectal biopsy sample
Ctrl5	50-60	Male	Rectal biopsy sample
Ctrl6	40-50	Male	Rectal biopsy sample
Ctrl7	50-60	Male	Rectal biopsy sample
Ctrl8	40-50	Male	Rectal biopsy sample
Ctrl9	40-50	Male	Rectal biopsy sample
Ctrl10	40-50	Male	Rectal biopsy sample
Ctrl11	50-60	Male	Rectal biopsy sample
Ctrl12	50-60	Male	Rectal biopsy sample
Ctrl13	30-40	Male	Rectal biopsy sample
Ctrl14	50-60	Male	Rectal biopsy sample
Ctrl15	30-40	Female	Rectal biopsy sample
Ctrl16	30-40	Female	Rectal biopsy sample
Ctrl17	40-50	Female	Rectal biopsy sample
Ctrl18	40-50	Female	Rectal biopsy sample
Ctrl19	40-50	Female	Rectal biopsy sample
Ctrl20	40-50	Female	Rectal biopsy sample
Ctrl21	50-60	Female	Rectal biopsy sample
Ctrl22	50-60	Female	Rectal biopsy sample
Ctrl23	60-70	Female	Rectal biopsy sample
Ctrl24	30-40	Female	Rectal biopsy sample
Ctrl25	40-50	Female	Rectal biopsy sample
Ctrl26	40-50	Female	Rectal biopsy sample
Ctrl27	50-60	Female	Rectal biopsy sample
Ctrl28	30-40	Female	Rectal biopsy sample
Ctrl29	30-40	Female	Rectal biopsy sample

Supplementary Table 2. Primers used in the study

Target	Primer Sequence
m-S100a8-F	AAATCACCATGCCCTCTACAAG
m-S100a8-R	CCCACTTTTATCACCATCGCAA
m-S100a9-F	ATACTCTAGGAAGGAAGGACACC
m-S100a9-R	TCCATGATGTCATTTATGAGGGC
m-Reg3g-F	GATGCCCCATCTTCACGTAGCA
m-Reg3g-R	GAGCACAGACACAAGATGTCCTGAG
m-Reg3b-F	ACTCCCTGAAGAATATACCCTCC
m-Reg3b-R	CGCTATTGAGCACAGATACGAG
mt-Nd3-F	TAGTTGCATTCTGACTCCCCCA
mt-Nd3-R	GAGAATGGTAGACGTGCAGAGC
mt-Nd4-F	CGCCTACTCCTCAGTTAGCCA
mt-Nd4-R	TGATGTGAGGCCATGTGCGA
mt-Co1-F	TCAACATGAAACCCCCAGCCA
mt-Co1-R	GCGGCTAGCACTGGTAGTGA
mt-Co2-F	ACCTGGTGAACTACGACTGCT
mt-Co2-R	TCCTAGGGAGGGGACTGCTC
mt-Co3-F	CCAAGGCCACCACACTCCTA
mt-Co3-R	GGTCAGCAGCCTCCTAGATCA
mt-Atp6-F	AGCTCACTTGCCCACTTCCT
mt-Atp6-R	AAGCCGGACTGCTAATGCCA
mt-Nd1-F	GCTTTACGAGCCGTAGCCCA
mt-Nd1-R	GGGTCAGGCTGGCAGAAGTAA
mt-Nd2-F	CCTCCTGGCCATCGTACTCA
mt-Nd2-R	GAATGGGGCGAGGCCTAGTT
m-Tfb1m-F	GGCTGAGAGACTTGTAGCCACT
m-Tfb1m-R	AGGTGCACCACTCCTACATCAA
m-Tfb2m-F	TTTGGCAAGTGGCCTGTGAC
m-Tfb2m-R	ACTGATTCCCCGTGCTTTGACT
m-Tfam-F	GAGCAGCTAACTCCAAGTCAG
m-Tfam-R	GAGCCGAATCATCCTTTGCCT
Tfam-ChIP-F	ATGAGGCTATAACTAATA
Tfam-ChIP-R	TTATCCACTAAAACTGTC
m-Xiap-F	CGAGCTGGGTTTCTTTATACCG
m-Xiap-R	GCAATTTGGGGATATTCTCCTGT
m-Ccl4-F	TTCCTGCTGTTTCTCTTACACCT
m-Ccl4-R	CTGTCTGCCTCTTTTGGTCAG
m-Cflip-F	GCTCCAGAATGGGCGAAGTAA
m-Cflip-R	ACGGATGTGCGGAGGTAAAAA
m-Npm1-F	ATGGAAGACTCGATGGATATGGA
m-Npm1-R	ACCGTTCTTAATGACAACTGGTG
m-Tjp1-F	GCTTTAGCGAACAGAAGGAGC
m-Tjp1-R	TTCATTTTTCCGAGACTTCACCA

m-Tjp2-F	GTTTGCCGTTCAGCAGCTTAG
m-Tjp2-R	CTTCAAAACCTCGGTCGTCAT
m-Cldn2-F	CAACTGGTGGGCTACATCCTA
m-Cldn2--R	ATCCAGAGGCCCTTGGAAAAG
m-Cldn3--F	ACCAACTGCGTACAAGACGAG
m-Cldn3--R	CGGGCACCAACGGGTTATAG
m-Stat3-F	CAATACCATTGACCTGCCGAT
m-Stat3-R	GAGCGACTCAAACTGCCCT
m-IRF1-F	ATGCCAATCACTCGAATGCG
m-IRF1-R	TTGTATCGGCCTGTGTGAATG
m-Gata3-F	CTCGGCCATTCGTACATGGAA
m-Gata3-R	GGATACCTCTGCACCGTAGC
