# Supplementary material for: Nucleophosmin 1 promotes mucosal immunity by supporting mitochondrial oxidative phosphorylation and ILC3 activity
Source: Nat Immunol. 2024 Aug 5;25(9):1565–79. doi: 10.1038/s41590-024-01921-x (PMC11362010; doi:10.1038/s41590-024-01921-x)
Supplement: Supplementary file 2 — Reporting Summary [file 41590_2024_1921_MOESM2_ESM.pdf]

nature portfolio

Corresponding author(s): Changgeng Peng, Shiyang Li and Minxuan Sun

Last updated by author(s): Jul 4, 2024

Reporting Summary

Nature Portfolio wishes to improve the reproducibility of the work that we publish. This form provides structure for consistency and transparency in reporting. For further information on Nature Portfolio policies, see our Editorial Policies and the Editorial Policy Checklist.

Statistics

For all statistical analyses, confirm that the following items are present in the figure legend, table legend, main text, or Methods section.

n/a	Confirmed	
☐	☒	The exact sample size (*n*) for each experimental group/condition, given as a discrete number and unit of measurement
☐	☒	A statement on whether measurements were taken from distinct samples or whether the same sample was measured repeatedly
☐	☒	The statistical test(s) used AND whether they are one- or two-sided<br>*Only common tests should be described solely by name; describe more complex techniques in the Methods section.*
☐	☒	A description of all covariates tested
☐	☒	A description of any assumptions or corrections, such as tests of normality and adjustment for multiple comparisons
☐	☒	A full description of the statistical parameters including central tendency (e.g. means) or other basic estimates (e.g. regression coefficient) AND variation (e.g. standard deviation) or associated estimates of uncertainty (e.g. confidence intervals)
☐	☒	For null hypothesis testing, the test statistic (e.g. *F*, *t*, *r*) with confidence intervals, effect sizes, degrees of freedom and *P* value noted<br>*Give P values as exact values whenever suitable.*
☒	☐	For Bayesian analysis, information on the choice of priors and Markov chain Monte Carlo settings
☒	☐	For hierarchical and complex designs, identification of the appropriate level for tests and full reporting of outcomes
☐	☒	Estimates of effect sizes (e.g. Cohen's *d*, Pearson's *r*), indicating how they were calculated

Our web collection on statistics for biologists contains articles on many of the points above.

Software and code

Policy information about availability of computer code

Data collection	Flow cytometric analysis was performed using the BD FACSCelesta Flow Cytometer and Gallios Flow Cytometer.<br>Immunofluorescence: Leica SP5 confocal microscope.
Data analysis	Flow Cytometry data were analyzed by FlowJo V10, Immunofluorescence images were analyzed by Image J 64-bit Java 8. Statistical analyses were performed using GraphPad Prism V8. Data were presented as mean±SEM. Statistical significance was assessed by student's t-test (unpaired) or two-way ANOVA analyses. All statistical tests were two-tailed and a p-value of <0.05 was considered statistically significant.

For manuscripts utilizing custom algorithms or software that are central to the research but not yet described in published literature, software must be made available to editors and reviewers. We strongly encourage code deposition in a community repository (e.g. GitHub). See the Nature Portfolio guidelines for submitting code & software for further information.

Data

Policy information about availability of data

All manuscripts must include a data availability statement. This statement should provide the following information, where applicable:

- Accession codes, unique identifiers, or web links for publicly available datasets
- A description of any restrictions on data availability
- For clinical datasets or third party data, please ensure that the statement adheres to our policy

Smart-seq analysis of primary colonic ILC3 in Npm1+/+ and Npm1+/- mice can be assessed with GEO number “GSE271455”. scRNA-seq data of UC patients and healthy controls was downloaded from GSE182270 in the GEO repository. https://www.ncbi.nlm.nih.gov/geo/query/acc.cgi?acc=GSE182270.

Research involving human participants, their data, or biological material

Policy information about studies with human participants or human data. See also policy information about sex, gender (identity/presentation), and sexual orientation and race, ethnicity and racism.

Reporting on sex and gender	Male and female rectal tissue samples were used in our study, and this information is provided in Supplementary Table 1.
Reporting on race, ethnicity, or other socially relevant groupings	We worked to ensure sex and gender balance in the recruitment of participants.<br>We worked to ensure racial and ethnic or other types of diversity in the recruitment of participants.
Population characteristics	Information about the human rectal tissue samples is provided in Supplementary Table 1.
Recruitment	Human rectal tissue samples were obtained from approved tissue banks. No patients were recruited.
Ethics oversight	Pathological sections were obtained from ulcerative colitis patients, Crohn's disease patients and healthy individuals after approval had been obtained from Ethics Committee of Shandong University School of Basic Medicine (ECSBMSSDU2020-1-035).

Note that full information on the approval of the study protocol must also be provided in the manuscript.

Field-specific reporting

Please select the one below that is the best fit for your research. If you are not sure, read the appropriate sections before making your selection.

☒ Life sciences ☐ Behavioural & social sciences ☐ Ecological, evolutionary & environmental sciences

For a reference copy of the document with all sections, see nature.com/documents/nr-reporting-summary-flat.pdf

Life sciences study design

All studies must disclose on these points even when the disclosure is negative.

Sample size	No statistical method was used to predetermine sample size. All studies using at least 3-5 animals per group, all experiments were performed at least twice to ensure reproducibility, which is commonly accepted in the field of immunology.
Data exclusions	No sample were excluded from analysis.
Replication	All attempts at replication were successful. All experiments were independently performed at least twice to ensure reproducibility.
Randomization	For DSS/TNBS/AOM-DSS animal studies to assess the changes of Npm1-deficiency, no method of randomization was used. Mice were grouped according to genotype and all experiments were performed with sex-matched littermates. For bezafibrate experiment, mice were first grouped by genotype and than randomly assigned to two groups (bezafibrate treated group and control group). For CD11b/CD3 antibody treatment experiments, mice were first grouped by genotype and than randomly assigned to two groups (CD11b/CD3 antibody treated group and IgG antibody treated group).
Blinding	Animal studies were not blinded (mice were named with mouse ID and genotyped within 6 weeks of birth). Group allocation was not applicable because mice were grouped based on and compared across different genotypes. Histological analysis were analyzed by two independent investigators, who had limited knowledge of the group of mice and patients. DAI were analyzed by two independent investigators, who had limited knowledge of the group of mice and patients. Data of FACS, qPCR, ELISA, et.al were collected by an investigator with only the knowledge of mouse ID (without grouping information).

Reporting for specific materials, systems and methods

We require information from authors about some types of materials, experimental systems and methods used in many studies. Here, indicate whether each material, system or method listed is relevant to your study. If you are not sure if a list item applies to your research, read the appropriate section before selecting a response.

Materials & experimental systems

n/a	Involved in the study
☐	☒ Antibodies
☐	☒ Eukaryotic cell lines
☒	☐ Palaeontology and archaeology
☐	☒ Animals and other organisms
☒	☐ Clinical data
☒	☐ Dual use research of concern
☒	☐ Plants

Methods

n/a	Involved in the study
☒	☐ ChIP-seq
☐	☒ Flow cytometry
☒	☐ MRI-based neuroimaging

Antibodies

Antibodies used

Anti-mouse CD45 eFlour 506 (clone 30-F11) eBioscience Cat#69-0451-82; RRID:AB_2637147 dilution 1:100
Anti-mouse RORγt PE (clone B2D) eBioscience Cat#12-6981-80; RRID:AB_10807092 dilution 1:50
Anti-mouse Ly-6G PE (clone 1A8-Ly6g) eBioscience Cat#12-9668-80; RRID:AB_2572720 dilution 1:100
Anti-mouse CD127 Super Bright 645 (clone A7R34) eBioscience Cat#64-1271-80; RRID:AB_2744868 dilution 1:100
Anti-mouse F4/80 FITC (clone BM8) eBioscience Cat#11-4801-82; RRID:AB_2637191 dilution 1:100
Anti-mouse CD3 Alexa-488 (clone 17A2) eBioscience Cat#53-0032-82; RRID:AB_2848414 dilution 1:100
Anti-mouse CD34 FITC (clone RAM34) eBioscience Cat#11-0341-81; RRID:AB_465021 dilution 1:100
Anti-mouse CD117 APC (clone ACK2) eBioscience Cat#17-1172-82; RRID:AB_469433 dilution 1:100
Anti-mouse CD19 eFlour 450 (clone 1D3) eBioscience Cat#48-0193-82; RRID:AB_2734905 dilution 1:100
Anti-mouse IL-22 PE (clone 1H8PWSR) eBioscience Cat#12-7221-80; RRID:AB_10597428 dilution 1:50
Anti-mouse CD16/32 (clone 93) eBioscience Cat#14-0161-82; RRID:AB_467133 dilution 1:100
Anti-mouse CD4 APC (clone V4) Biolegend Cat#100411; RRID; AB_312696 dilution 1:100
Anti-mouse IL-17A BV421 (clone TC11-18H10) BD Cat#566286; RRID; AB_2687547 dilution 1:50
Anti-mouse Lineage Percp-cy5.5 Cocktail BD Cat#51-9006964; RRID:AB_10612020 dilution 1:50
Anti-mouse T-bet PE (clone 4B10) eBioscience Cat#25-5825-82; RRID:AB_10565980 dilution 1:100
Anti-mouse IFNgamma-APC (clone XMG1.2) eBioscience Cat#17-7311-82; RRID:AB_469504 dilution 1:50
Anti-mouse NKp46-PerCPcy5.5 (clone 29A1.4) eBioscience Cat#46-3351-82; RRID:AB_1834441 dilution 1:50
Anti-mouse FOXP3-eFlour 450 (clone FJK-16S) eBioscience Cat#48-5773-82; RRID:AB_1518812 dilution 1:100
Anti-mouse CCR6-BV421 (clone 29-2L17) BioLegend Cat#129818; RRID:AB_11219003 dilution 1:50
Anti-mouse TCR gamma/delta-APC (clone GL3) BioLegend Cat#118116; RRID:AB_1731813 dilution 1:50
Anti-mouse CD127-FITC (clone A7R34) BioLegend Cat#135008; RRID:AB_AB_1937232 dilution 1:100
Rabbit SDHB Antibody Proteintech Cat# 10620-1-AP; RRID:AB_2285522 dilution 1:2000
Rabbit NDUFB8 Antibody Proteintech Cat# 14794-1-AP; RRID:AB_2150970 dilution 1:2000
Rabbit MT-ATP6 Antibody ABclonal Cat# A17960; RRID; AB_2861763 dilution 1:1000
Rabbit MT-CO1 Antibody ABclonal Cat# A17889; RRID; AB_2861744 dilution 1:1000
Rabbit UQCRC2 Antibody Proteintech Cat# 14742-1-AP; RRID:AB_2241442 dilution 1:1000
Rabbit NPM1 Antibody ABclonal Cat# A17983; RRID; AB_2861784 dilution 1:1000
Rabbit TOM20 Antibody Proteintech Cat# 66777-1-Ig; RRID:AB_2882123 dilution 1:200
Rabbit NF-kappaB p65 Antibody Cell Signaling Technology Cat# 8242; RRID; AB_10859369 dilution 1:1000
Mouse NF-kB p65/RelA Antibody ABclonal Cat# A10609 dilution 1:1000
Mouse NPM1 Antibody Proteintech Cat# 60096-1-Ig; RRID:AB_2155162 dilution 1:200
ROR gamma (t) Monoclonal Antibody (clone AFKJS-9), eBioscience Cat#14-6988-82; RRID:AB_1834475 dilution 1:200
Donkey anti-Mouse IgG (H+L) Highly Cross-Adsorbed Secondary Antibody, Alexa Fluor™ 488 Invitrogen Cat#A21202; RRID:AB_141607 dilution 1:2000
Donkey anti-Rat IgG (H+L) Highly Cross-Adsorbed Secondary Antibody, Alexa Fluor™ 555 Invitrogen Cat#A78945; RRID:AB_2910652 dilution 1:2000
Donkey anti-Rabbit IgG (H+L) Highly Cross-Adsorbed Secondary Antibody, Alexa Fluor™ 647 Invitrogen Cat#A31573; RRID: AB_2536183 dilution 1:2000
IRDye® 680RD Donkey anti-Rabbit IgG Secondary Antibody, LICORbio, Cat#926-68073; RRID: AB_2716687 dilution: 1:10000
IRDye® 800CW Donkey anti-Mouse IgG Secondary Antibody, LICORbio, Cat#926-32212; RRID: AB_2716622 dilution: 1:10000

Validation

All antibodies listed above were commercially available and validated by the manufacturer. Validation data are available on the manufacturer's website. All antibodies described here have been further optimised for an appropriate concentration by testing several dilutions.

Eukaryotic cell lines

Policy information about cell lines and Sex and Gender in Research

Cell line source(s)

Mouse MNK3 cell line; BLUEFBIO Cat# BFN60807579
Human HEK293T cell line; Lab preserve

Authentication

Identity of the cell lines were frequently checked by their morphological features.

Mycoplasma contamination	All cell lines were tested to be mycoplasma-negative by the standard PCR method.
Commonly misidentified lines (See ICLAC register)	No commonly misidentified cell lines are used in this study.

Animals and other research organisms

Policy information about studies involving animals; ARRIVE guidelines recommended for reporting animal research, and Sex and Gender in Research

Laboratory animals	Npm1+/-, Npm1UTR+/-, Npm1flox/+ mice were generated in this study by CRO company Shanghai Model Organisms Center, Inc.<br>Villin cre/+ mice and Rorc cre/+ mice were gifted by Li Lab from Shandong University.<br>Apc min/+ mice were purchased from The Jackson Laboratory, Cat# 002020<br>All experiments were performed using C57BL/6J mice, which also served as controls for Npm1+/-, Npm1UTR-/- and Apcmin/+ mice.<br>Npm1flox/flox mice were served as controls for Rorccre/+Npm1flox/flox and Villincre/+Npm1flox/flox mice.<br>6-8 week-old male mice were used for experiments.<br>Mice were housed in individually ventilated cages under 12h light-12h dark cycle with normal food and water.
Wild animals	No wild animals were included in this study.
Reporting on sex	All the mice used in the experiments were male.
Field-collected samples	No field-collected samples included in this study
Ethics oversight	All animal experiments were approved and are in accordance with the Institutional Animal Care and Use Committee guidelines at Suzhou Institute of Biomedical Engineering and Technology (2021-C058) and Shandong University (ECSBMSSDU2020-2-057).

Note that full information on the approval of the study protocol must also be provided in the manuscript.

Flow Cytometry

Plots

Confirm that:

☒ The axis labels state the marker and fluorochrome used (e.g. CD4-FITC).

☒ The axis scales are clearly visible. Include numbers along axes only for bottom left plot of group (a 'group' is an analysis of identical markers).

☒ All plots are contour plots with outliers or pseudocolor plots.

☒ A numerical value for number of cells or percentage (with statistics) is provided.

Methodology

Sample preparation	To isolate leukocytes from the lamina propria, we incubated intestinal segments of approximately 0.5 cm at 37°C for 1.5 hours in complete RPMI medium, supplemented with DNase I (150 μg/ml, Sigma) and collagenase VIII (300 U/ml, Sigma). The digested fragments were triturated and filtered through a 100 μm cell strainer. The cells were collected from the interface of the 80% and 40% Percoll gradients after centrifugation at 660 xg for 15 minutes at room temperature. Prior to surface staining, Fc receptors were blocked using CD16/32 antibody (eBioscience). Leukocytes isolated from the intestinal lamina propria were then stained with antibodies against the distinct markers. For cytokine staining, cells were stimulated with PMA (50 ng/ml) and ionomycin (500 ng/ml) for 2 hours, along with the addition of brefeldin A (2 μg/ml). Live and dead cells were distinguished using the Live and Dead Violet Viability Kit (BioLegend).
Instrument	Flow cytometric analysis was performed using the BD FACSCelesta Flow Cytometer and Gallios Flow Cytometer. Samples for RNAseq and in vivo experiment were sorted on Moflo Astrios EQ and BD FACSMelody Flow Cytometer.
Software	FlowJo software v10
Cell population abundance	Purity above 90%
Gating strategy	Live single cells were identified based on FSC/SSC as well as live/dead cell staining. The sorted ILC3s were defined as Live+Lin-CD45lowCD90.2high cells. For analysis, macrophages were gated as CD45+CD11b+F4/80+ cells; neutrophils were gated as CD45+CD11b+Ly6G+ cells; eosinophils were gated as CD45+CD11b+SIGLECF+; T cells were gated as CD45+CD3+; B cells were gated as CD45+CD19+; ILC3s were gated as CD45+Lin-RORgt+ cells.

☒ Tick this box to confirm that a figure exemplifying the gating strategy is provided in the Supplementary Information.
