# Supplementary figures and images for: Nucleophosmin 1 promotes mucosal immunity by supporting mitochondrial oxidative phosphorylation and ILC3 activity
Source: Nat Immunol. 2024 Aug 5;25(9):1565–79. doi: 10.1038/s41590-024-01921-x (PMC11362010; doi:10.1038/s41590-024-01921-x)

Figure 3E mt-ATP6

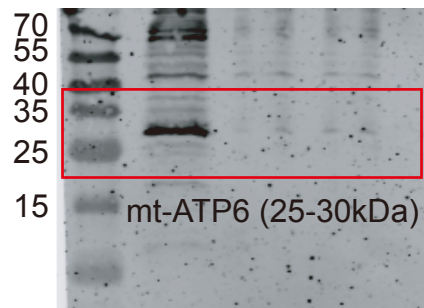

Figure 3E NDUFB8

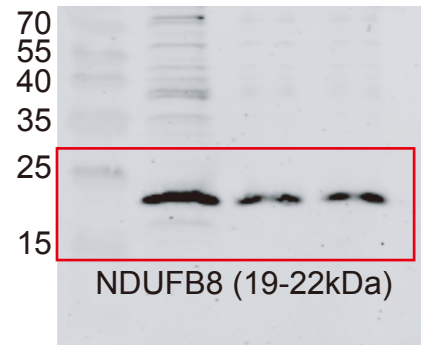

Figure 3E mt-CO1

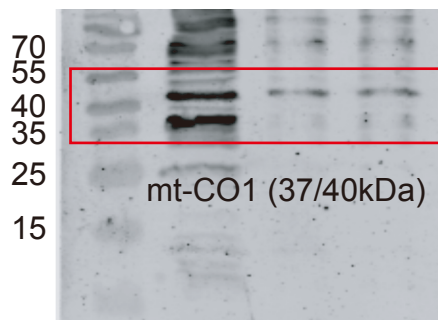

Figure 3E  $\beta$ -actin

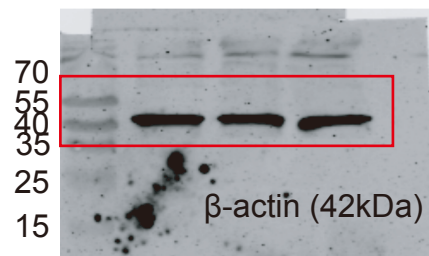

Figure 3E UQCRC2

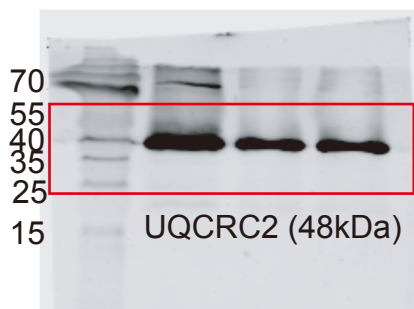

Figure 3E SDHB

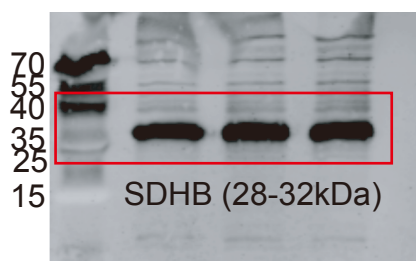

Supplement: Supplementary file 7 — Unprocessed western blots. [file 41590_2024_1921_MOESM7_ESM.pdf]

Figure 5D

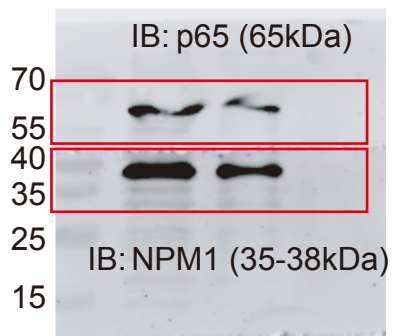

Figure 5F p65

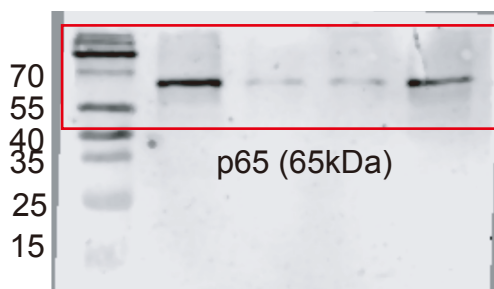

Figure 5F GAPDH

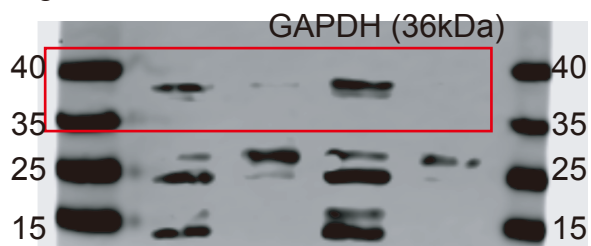

Figure 5F Histone-H3

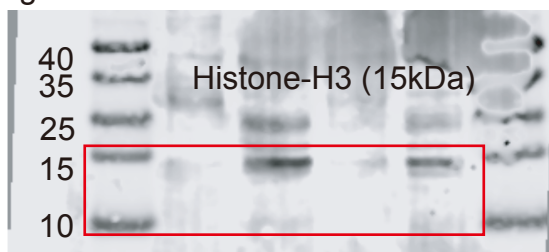

Supplement: Supplementary file 10 — Unprocessed western blots. [file 41590_2024_1921_MOESM10_ESM.pdf]

Extended Data Fig.1F NPM1

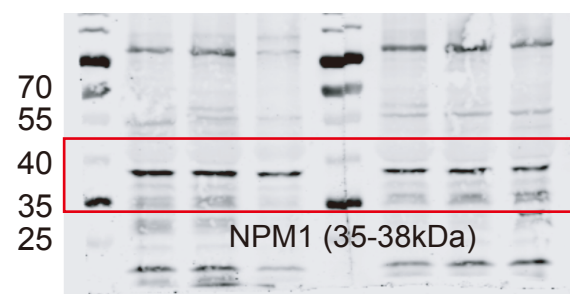

Extended Data Fig.1F Tubulin

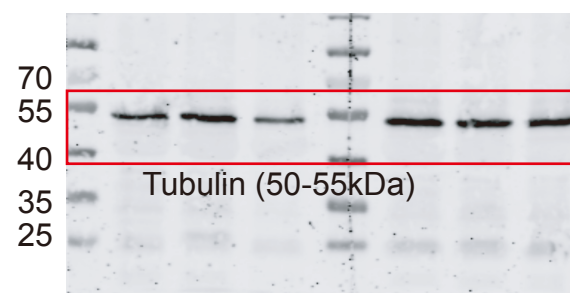

Supplement: Supplementary file 14 — Unprocessed western blots. [file 41590_2024_1921_MOESM14_ESM.pdf]
